# Application of Circulating Tumor DNA as a Biomarker for Non-Small Cell Lung Cancer

**DOI:** 10.3389/fonc.2021.725938

**Published:** 2021-08-05

**Authors:** Jialiang Yang, Yan Hui, Yanxiang Zhang, Minghui Zhang, Binbin Ji, Geng Tian, Yangqiang Guo, Min Tang, Lianxing Li, Bella Guo, Tonghui Ma

**Affiliations:** ^1^Chifeng Municipal Hospital, Chifeng, China; ^2^Qingdao Geneis Institute of Big Data Mining and Precision Medicine, Qingdao, China; ^3^Geneis Beijing Co., Ltd., Beijing, China; ^4^China National Intellectual Property Administration, Beijing, China; ^5^School of Life Sciences, Jiangsu University, Zhenjiang, China; ^6^Genetron Health (Beijing) Co. Ltd., Beijing, China

**Keywords:** non-small cell lung cancer, circulating tumor DNA, molecular testing, liquid biopsy, immunotherapies, therapeutic response

## Abstract

**Background:**

Non-small cell lung cancer (NSCLC) is one of the most prevalent causes of cancer-related death worldwide. Recently, there are many important medical advancements on NSCLC, such as therapies based on tyrosine kinase inhibitors and immune checkpoint inhibitors. Most of these therapies require tumor molecular testing for selecting patients who would benefit most from them. As invasive biopsy is highly risky, NSCLC molecular testing based on liquid biopsy has received more and more attention recently.

**Objective:**

We aimed to introduce liquid biopsy and its potential clinical applications in NSCLC patients, including cancer diagnosis, treatment plan prioritization, minimal residual disease detection, and dynamic monitoring on the response to cancer treatment.

**Method:**

We reviewed recent studies on circulating tumor DNA (ctDNA) testing, which is a minimally invasive approach to identify the presence of tumor-related mutations. In addition, we evaluated potential clinical applications of ctDNA as blood biomarkers for advanced NSCLC patients.

**Results:**

Most studies have indicated that ctDNA testing is critical in diagnosing NSCLC, predicting clinical outcomes, monitoring response to targeted therapies and immunotherapies, and detecting cancer recurrence. Moreover, the changes of ctDNA levels are associated with tumor mutation burden and cancer progression.

**Conclusion:**

The ctDNA testing is promising in guiding the therapies on NSCLC patients.

## Introduction

**Figure 1:**
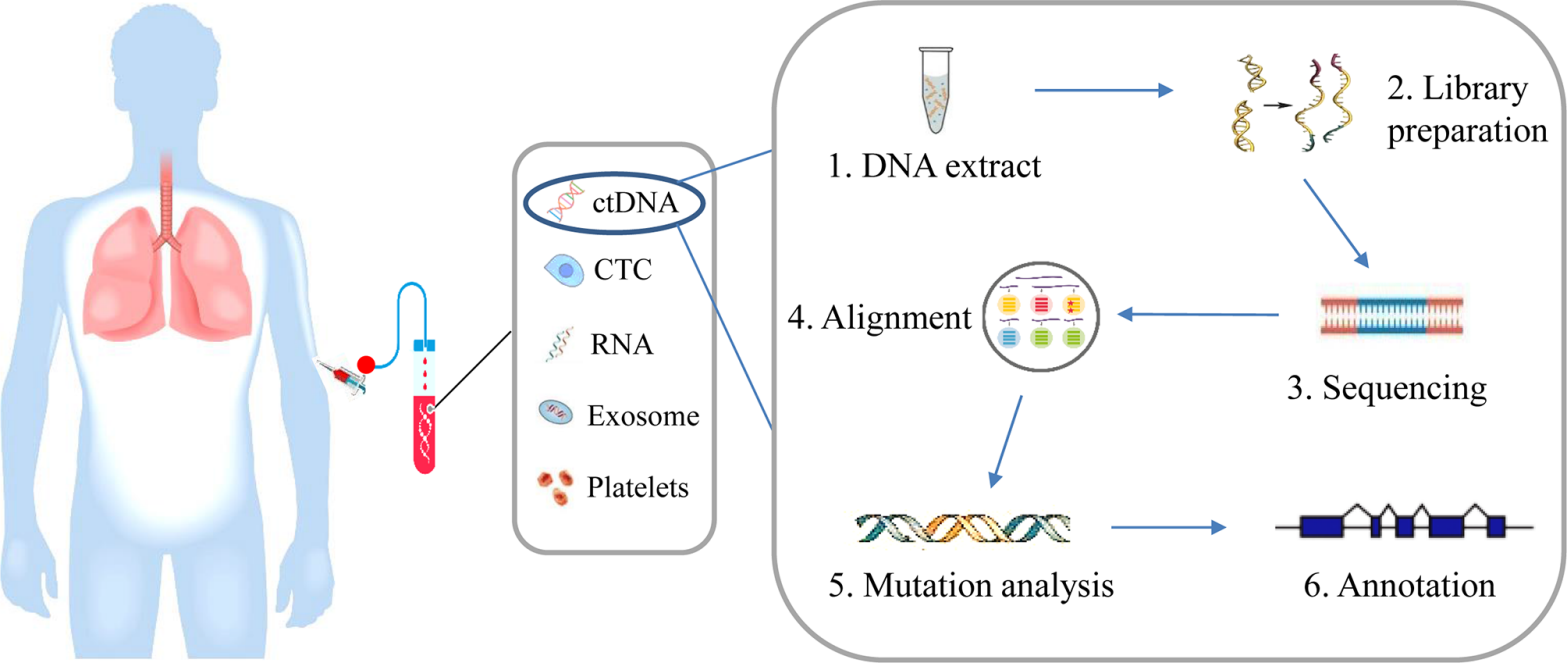
Overview of liquid biopsy. Blood is sampled from patients, which contains ctDNA, CTC, RNA, exosome, and tumor-educated platelets. CtDNA is extracted from blood plasma and gene variation can be analyzed by next generation sequencing involving a few steps, including DNA extraction, DNA library preparation, sequencing, sequence alignment, mutation annotation, and so on.

Liquid biopsy refers most often to the analysis of tumor-derived materials, including circulating tumor cell (CTC), circulating tumor DNA (ctDNA), circulating tumor RNA (ctRNA), exosome, and tumor-educated platelet (TEP) from blood plasma (**Figure 1**). CTCs are released from tumor tissue; ctDNA is secreted from apoptotic or necrotic tumor cells; and exosomes are membrane-bound vesicles released from tumor cells. Until now, ctDNA is the only circulating biomarker approved for selecting patients with targeted therapy, whereas other liquid biopsy sources, such as CTCs, RNA, exosomes, and TEP, are still at clinical research phase. Therefore, this review will focus on the interpretation of ctDNA testing and its potential clinical applications.

As reported by Mandel and Metais for the first time in 1948, circulating cell-free DNA (cfDNA) refers to double-stranded DNA fragments in liquid biopsy with lengths close to or lower than 200 base pair (bp) (1). CfDNA is present at low levels in the plasma of healthy persons, but at high levels in those of cancer patients (2, 3). CfDNA of tumor origin is referred to as ctDNA. Recent studies have confirmed that the fraction of ctDNA in total cfDNA greatly varied in cancer patients. Patients with early stage tumors present lower fractions of ctDNA than those in the advanced stage (4–6). Although the concentration of ctDNA has been suggested to predict the outcomes of patients with non-small cell lung cancer (NSCLC), the criterion for appropriate cutoffs is unavailable in clinical utility (7). However, even in the same cancer type, substantial variability has been observed indifferent patients (4, 8, 9). Mutational analysis of ctDNA has already been proven to be promising in early cancer detection and cancer recurrence evaluation (10–15).

In the last decade, important advancements have been achieved in NSCLC (16, 17). For example, small molecule tyrosine kinase inhibitors (TKIs) were proven to be effective for patients with advanced lung adenocarcinoma who harbor somatic mutation of epidermal growth factor receptor (EGFR), as well as the rearrangement of echinoderm microtubule-associated protein-like 4 (EML4) with anaplastic lymphoma (ALK) (18–22). More recently, immune checkpoint inhibitors (ICI) therapies have shown significant benefit in the treatment of patients with NSCLC (16, 23). Besides programmed cell death 1 (PD-1) and programmed cell death ligand 1 (PD-L1), tumor mutation burden (TMB) is a promising biomarker in predicting the outcomes of NSCLC patients with immunotherapy (24–27). Therefore, the accurate information of a cancer patient’s genetic status is critical in guiding personalized medication. Although tissue biopsy remains the gold standard for molecular testing of cancers, it presents some disadvantages in clinical situations. First, tumor biopsy is highly risky because of its invasive nature and is dangerous for extremely serious patients. Second, tumor biopsy is inappropriate in a few occasions like cancer early detection and recurrence surveillance, where multiple testing at different time points is necessary. Finally, tumor biopsy can only examine one tissue at a time and usually cannot reflect the mutational landscape of the whole body (28–31).

CtDNA testing provides a powerful and effective alternative method to tissue biopsy for cancer diagnosis, treatment, and prognosis. In the past 5 years, a high concordance has been confirmed between plasma and tissue samples, further encouraging the exploration of ctDNA in clinical applications (30, 32–34). Some studies have suggested that ctDNA levels can be used to monitor the response of patients to local and systemic therapies (35–37). Additionally, ctDNA testing can effectively predict responses to targeted therapies in multiple tumor types (4, 38–41). Despite the important achievements in ctDNA, there is no systematic review comprehensively introducing recent development in clinical applications of ctNDA as a biomarker in NSCLC, to our best knowledge.

**Figure 2:**
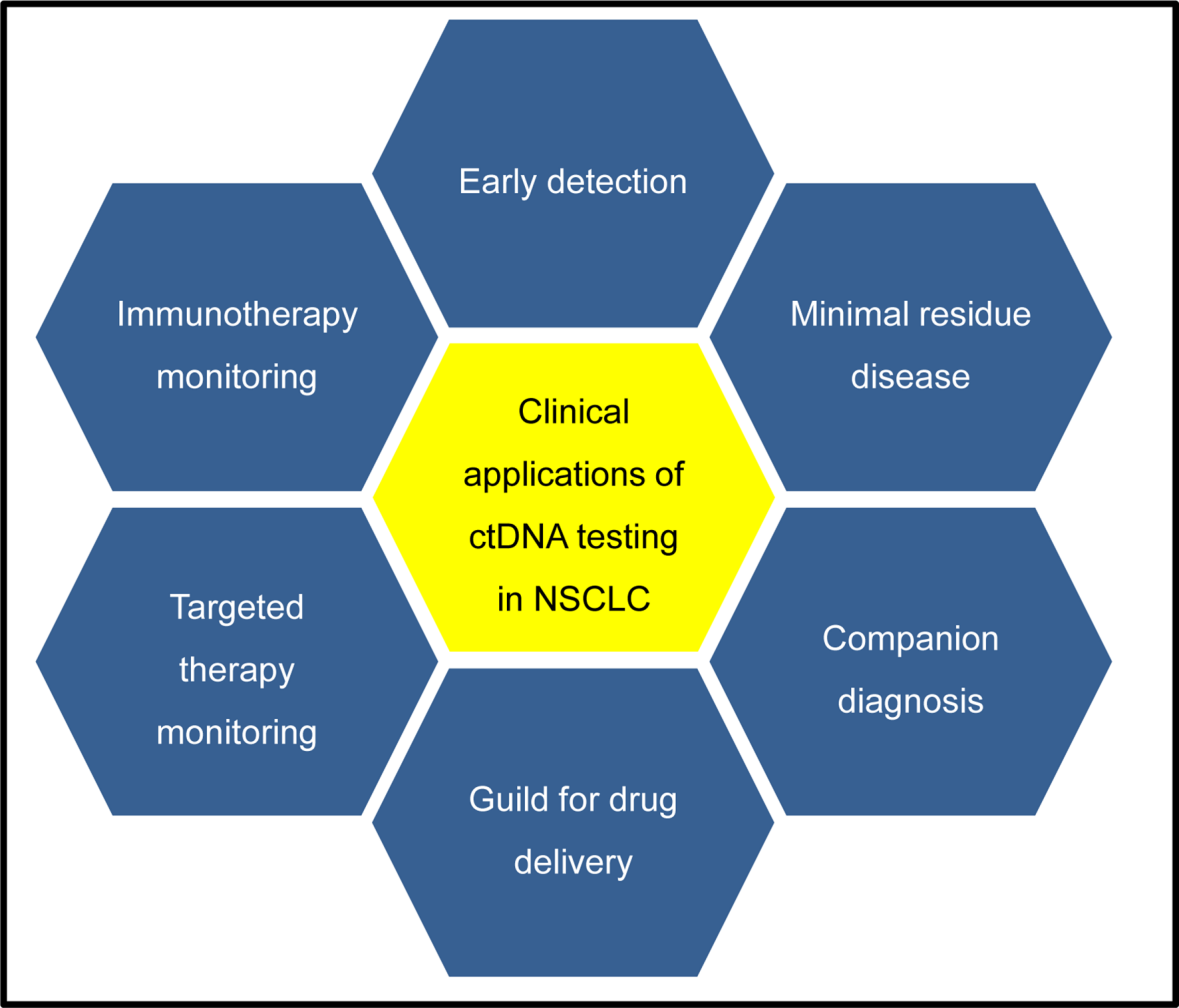
Potential clinical applications of ctDNA testing in NSCLC patients. CtDNA testing in early tumors detection are under development, which may identify patients with cancers at early stage when they are more likely to be curable. CtDNA testing in minimal residual disease detection after surgery can provide the evidence of tumor relapse, which may offer an opportunity for early intervention for patients according to risk of recurrence. For companion diagnosis, ctDNA testing is available to identify many somatic alterations, which can provide a guide for treatment decisions for patients with targeted therapies. Analysis of cfDNA has been used to monitor the response to targeted or immunotherapy, which can provide a molecular basis to guide the subsequent therapy choice.

In this review, we aim to describe the potential clinical application of ctDNA testing, from aiding cancer diagnosis to guiding patients’ treatment and detection of minimal residual disease, and to monitoring the response to cancer treatment in a dynamic way (**Figure 2**). Finally, a few promising approaches are highlighted, which may become available in a wide range of clinical applications in the future.

## Approaches of CtDNA Testing

Since cfDNA is derived from various cells and rapidly cleared in the circulation system, it usually presents in body fluid in a short time and with a limited level. Hence, it is critical to optimize experimental techniques for cfDNA (ctDNA) isolation and analysis. For cfDNA isolation, the anticoagulant EDTA can stabilize cfDNA and prevent the contamination with germline DNA released from normal blood cells. However, blood samples have to be processed by methods like leukocyte fixation within 6 h after collection. Leukocyte fixation allows for easy shipping and centralized processing, which can stabilize cfDNA and normal blood cells for two days (42). A recent study reported that specialized cfDNA collection tube with the stabilization reagent provides even higher flexibility for sample processing, i.e., up to 14 days without affecting cfDNA detection (43).

The fraction of ctDNA in total cfDNA can vary greatly form less than 0.1% to more than 90% (4). Several high-sensitivity approaches are available to analyze ctDNA even at low levels (**Table 1**), including peptide nucleic acids (PNA)-based methods (44), quantitative polymerase chain reaction (qPCR) and droplet digital PCR (ddPCR), beads emulsion amplification and magnetics (BEAMing) (45). However, those methods are not adequate to analyze multiple genes through a high-throughput screening. Several targeted next-generation sequencings (NGS) have been developed for ctDNA testing, including tagged amplicon-based sequencing (TAm-Seq) (46), cancer personalized profiling by deep sequencing (CAPP-Seq) (36), the targeted error correction sequencing (TEC-Seq) (47). TAm-Seq includes two steps of amplification. The first step of amplification is performed to capture the starting molecules present in the template by all primers, then, the second step of amplification is followed with a limited couple of primers in the access array. This approach is only detected in single-nucleotide variants (SNV), insertion, or deletions (indels) (46). CAPP-Seq is a capture-based NGS ctDNA detection method, the defined mutated regions are hybridized by the biotinylated probes. Several types of somatic mutations could be detected by CAPP-Seq, including SNV, indels, copy number alterations (CNAs), and rearrangements (36). TEC-Seq is based on targeted capture of multiple regions of the genome and deep sequencing of DNA fragments, which allow sensitive and specific detection of low abundance sequence alterations (47). Whole-exome sequencing or whole-genome sequencing with deep depth can provide a more comprehensive profiling of ctDNA (12, 48). However, because of the high costs and large volume of blood per patient, the application of these approaches to patients with advanced lung cancer is limited.

**Table 1:** A summary of methods for detection of genetic alterations in cfDNA and their performances.

Test	Technology	Limit of detection	Type of variants
Candidate variants analysis	qPCR	0.05-0.1%	SNVs, indels
	PNA-based methods	0.1%	
	BEAMing	0.001-0.1%	
	ddPCR	0.001-0.1%	
Next-generation sequencing	TAm-Seq	0.2-2%	SNVs, indels
	TEC-Seq	<0.01%	
	CAPP-Seq	0.01%	SNVs, indels, CNVs and fusions
	Whole Exome sequencing	5-10%	
	Whole Genome sequencing	1-10%	

## CtDNA Testing for Companion Diagnosis

Tumor molecular testing has become a fundamental practice to guide the selection of therapy in cancer medicine. Tissue biopsy remains the gold standard for patients with lung cancer diagnosis. However, invasive biopsy can be high risk, and patients would prefer liquid biopsy. This makes ctDNA testing an attractive tool, which could replace tissue biopsy in some specific situations. Although previous studies revealed a low concordance between the somatic variations detected from plasma and those detected from tissue samples (49, 50). For example, Torga and Pienta observed big differences in somatic mutations called from two different commercial ctDNA testing assays and those from tissue samples in 40 patients with metastatic prostate cancer (51). However, these studies may have bias. For example, tumor samples were collected at baseline time, whereas plasma samples were collected during the therapy. In addition, the enrolled patients had received therapy, which might lead to the alteration of mutation profiles. As a result, the discordance might be caused at least in partial by inappropriate experimental designs.

In the past years, a few larger and more carefully enrolled studies have indicated a relative high concordance between plasma and tissue samples obtained simultaneously, encouraging the clinical usage of ctDNA testing (30, 32–34). Recently, Roche’s Cobas plasma EGFR mutation test v2 was approved as the first ctDNA-testing tool by the Food and Drug Administration (FDA), which paved the way of liquid biopsy in clinical applications. Cobas plasma EGFR mutation test v2 can identify multiple mutations in exons 18, 19, 20, and 21 of *EGFR* in NSCLC patients, including exon 19 deletion, and L858R, G719X, S768I, L861Q, and T790M substitution. Among those, exon 19 deletion and L858R mutation are related to the increase of sensitivity to tyrosine kinase inhibitors (52). However, T790M mutation is often resistant to tyrosine kinase inhibitors, such as erlotinib, gefitinib, and afatinib, whereas it responds to osimertinib (29, 53). Hence, the test can guide the usage of tyrosine kinase inhibitors in treating NSCLC patients (54).

In addition to checking *EGFR* mutational status, ctDNA testing can also be used to detect mutations in other genes, such as *BRAF* mutations and *ALK* rearrangements. It has been indicated that NSCLC patients harboring *BRAF* mutations have clinical benefits from targeted therapies (55). ctDNA testing has been indicated to be effective in identifying both *ALK* point mutations and fusions in lung cancer patients, who will most likely to respond to crizotinib and other ALK tyrosine kinase inhibitors (11, 56). To display comprehensive mutation profiles in patients with NSCLC, large gene panels were developed to identify oncogenes and tumor suppressor genes *via* ctDNA testing (10, 36). A recent prospective study demonstrated genetic variations in eight genes, including *BRAF*, *EGFR*, and *ERBB2* mutations, *ALK*, *RET*, and *ROS1* rearrangements, *MET* amplifications and exon 14 skipping, were recommended as biomarkers *via* the Guardant360 test based on eight 70-gene NGS panels in metastatic NSCLC (10). These genetic variations were detected with a high concordance rate between ctDNA testing and tissue genotyping, which even reached more than 98% when only the FDA-approved markers (i.e., *ALK*, *BRAF*, *EGFR*, and *ROS1*) were considered (10). Hence, these approaches can provide a guide for treatment decisions of patients with NSCLC with lots of targeted therapies available or in progress.

## CtDNA Testing for Detecting Minimal Residue Disease

Currently, there is a clinical challenge to determine which patients have a minimal residue disease after surgical resection, which may result in recurrence. However, adjuvant chemo-radiation therapy is not routinely used for cancer patients because of its toxic nature. Nowadays, serial computed tomography (CT) and positron emission tomography combined CT (PET/CT) imaging are used for surveilling advanced NSCLC patients after surgery or chemo-radiation therapy. However, identification of disease recurrence is always delayed by CT imaging because of the uncertain recurrent location and small size of tumor. Hence, liquid biopsy might provide an aid to predict the risk of disease recurrence.

The sensitivity of ctDNA testing relies upon the level of tumor DNA released into blood and collecting samples at optimal time. Several liquid biopsy approaches have been developed to identify low level of ctDNA even less than 0.01% of total cfDNA (37, 57). Indeed, the recent studies have demonstrated that ctDNA testing has an ability to detect minimal residue disease and tumor relapse after surgery therapy in several cancer types, including lung cancer (35, 58, 59). Interestingly, several key studies have shown that ctDNA testing can detect resistance mutations or disease progression prior to CT imaging (35, 60, 61).

The recent prospective study of the West Japan Oncology Group 8114LTR (WJOG8114LTR) evaluated the clinical significance of monitoring ctDNA in 57 patients with advanced lung adenocarcinoma harboring EGFR mutations during afatinib treatment. They indicated that a remarkable long progress-free survival (PFS) was observed in patients with undetectable EGFR mutations, whereas a short PFS was observed in patients with positive EGFR mutations (62). Similarly, another study has provided an evidence of ctDNA testing monitoring disease progression of patients with advanced NSCLC during treatment with erlotinib, and EGFR T790M mutation was detected by ctDNA testing earlier than clinically evident disease (60). Hence, ctDNA testing can monitor recurrence and save time for patients to receive available intervention and may prevent tumor cells spread and proliferation.

## CtDNA Testing for Therapeutic Response

For monitoring therapeutic response, a repeat or serial molecular testing is required after one or more lines of therapy. Patients would prefer minimally invasive ctDNA testing if it can provide an effective power to predict the treatment, other than repeat invasive tumor biopsies because of their high risks. CtDNA was released into blood can be triggered by tumor cell death. Hence, dynamic changes of ctDNA concentrations may predict the response to the treatment. Indeed, previous studies have indicated that ctDNA levels are associated with disease situation in patients with NSCLC patients during therapy. The decreasing ctDNA levels are related with response to therapy, whereas the increasing ctDNA levels are related with progressive disease (7, 35–37, 60). A complete response could be expected from undetectable levels of ctDNA after serial testing. Therefore, ctDNA testing may provide an aid to guide chemotherapy decision or dose-based radiation therapy.

Circulating tumor DNA testing has also been confirmed to be able to detect targetable mutations that are involved in driving acquired resistance to therapy (12, 38, 39). One of the earliest studies has illustrated the development of resistance to cancer therapy using a serial ctDNA testing. In particular, the authors demonstrated that the emergence of EGFR T790M gatekeeper mutation was detected by ctDNA testing in patients with NSCLC after gefitinib treatment. This finding supported the hypothesis of selective pressure resulted from therapy. Several studies have made efforts to identify the presence of EGFR T790M by ctDNA testing in NSCLC patients might benefit from osimertinib, a third-generation EGFR inhibitor (29, 53). Interestingly, acquired EGFR C797S mutation was identified by ctDNA testing from 15 patients with NSCLC harboring EGFR T790M mutation undergoing osimertinib treatment, illustrating a novel acquired resistance mechanism to this EGFR inhibitor (63). Remarkably, EGFR T790M and other EGFR mutations can be identified by Roche’s Cobas plasma EGFR mutation test v2 which has been approved by FDA as an aid to guide decisions of specific EGFR inhibitors (54). It paves the way to utilize the approach of liquid biopsy to monitor targeted therapy, whereas it is a limitation to track other genetic alterations besides of EGFR mutation. Therefore, a comprehensive ctDNA analysis is required for the identification of genetic alterations to guide in the response to targeted therapies.

Indeed, several studies revealed the EGFR-independent mechanism of primary or acquired resistance to treatments of EGFR inhibitors, a bypass signaling pathway activated by the occurrence of gene variations mutation, such as mutations in *BRAF*, *KRAS*, *PIK3CA*, and amplification of *MET* (64–70). In particularly, the last update of the phase III AURA3 trial (no. NCT02151981) showed that emergence of *MET* amplification is detected by ctDNA testing in advanced NSCLC patients with EGFR T790M mutation during osimertinib treatment, which indicated that *MET* amplification is one of resistance mechanisms to this treatment. To overcome resistance, several case reports observed the therapeutic efficacy of MET inhibitor combined with EGFR inhibitor in patients with lung cancer appearing *MET* amplification detected by ctDNA testing after resistance to EGFR inhibitor (71–73). Moreover, a key clinical trial investigated the combination of capmatinib with gefitinib applied in patients with NSCLC, acquiring *MET* amplification after failure of EGFR inhibitor therapy (74).

In addition to EGFR mutations, ctDNA testing was also used for monitoring response or resistance to ALK inhibitors therapy in patients with lung cancer *via* identification of appearance of *ALK* variations, including *ALK* point mutations and rearrangements (11, 56). For example, the recent study identified novel *ALK* point mutations by ctDNA testing at the progression line after advanced *ALK*-positive NSCLC patients resistant to crizotinib treatment, revealed the resistance mechanisms on disease progression (56).

## CtDNA Testing for Immunotherapy

Besides small molecule tyrosine kinase inhibitors therapy, immune checkpoint inhibitor (ICI) therapies have shown significant benefit in the treatment of different tumor types including NSCLC (16). However, only a subtype of NSCLC patients could benefit from ICI immunotherapies. In addition to PD-1 and PD-L1 biomarkers, tumor mutational burden (TMB) is a promising biomarker to predict clinical outcomes of NSCLC patients to ICI immunotherapies (24–26), and was approved by the FDA in 2020. Tissue biopsy remains the gold standard for molecular testing, whereas it is a clinical challenge to obtain adequate tumor tissue from advanced NSCLC patients by invasion biopsy. Thus, it needs to explore minimally invasive approach to aid in distinguishing patients who can benefit from ICI immunotherapies.

Nowadays, the concordance of blood TMB (bTMB) with tissue TMB (tTMB) has been confirmed by the whole-exon sequencing (WES) (75, 76). However, it is a challenge to apply WES approach in clinical routine, mainly because of the high gDNA inputs and high costs. Therefore, the feasibility of blood TMB analyzed by targeted NGS panels needs to be assessed. In a preliminary study, the authors used targeted NGS panels to assess the concordance of blood TMB (bTMB) with tissue TMB (tTMB) in the enrolled 97 patients, whereas the obtained correlation was unsatisfying due to the low concordance between bTMB and tTMB (77). A possible reason is the lack of standard method for TMB assessment in this study. For example, the Foundation-One panel was used for tissue TMB analysis, while the Guardant360 was used for blood TMB analysis. The different sequencing panels resulted in different coverage of genomic regions. Moreover, the mutation types used for TMB calculation were different in the studies. Furthermore, the cutoff of TMB values were varied to classify these patients. Therefore, the standard sequencing panels used for comparing bTMB and tTMB should be considered carefully in future studies.

Remarkably, the very recent studies evaluated the concordance of bTMB with tTMB in advanced NSCLC patients and revealed that bTMB estimated by ctDNA testing is feasible to predict the clinical outcomes of ICI immunotherapy (78, 79). For example, Wang et al. optimized gene panel size and established 150 genes panel to estimate the bTMB value with response to ICI immunotherapies. The authors showed a high bTMB value of ≥ 6, which is related to long progression-free survival, suggesting that bTMB may be a promising biomarker to predict clinical benefit for advanced NSCLC patients to anti-PD-1 and anti-PD-L1 therapies (78). These studies provided the strong evidences to support bTMB determined by the targeted NGS panels. However, it still improves the sensitivity of ctDNA testing and develops a robust targeted NGS panels, and more clinical trials are required to confirm the abilities of bTMBas, a biomarker in liquid biopsy in the prediction of the clinical benefit of NSCLC patients from ICI immunotherapies.

Typically, a very recent study showed a set of neoantigens genes detected by personalized ctDNA testing, which can monitor the clinical response to ICI immunotherapies for advanced NSCLC patients (80). It is known that mutated neoantigens are the key targets of tumor-specific T-cells undergoing ICI immunotherapies. Therefore, the study focused on neoantigens-coding mutations detected by ctDNA testing during ICI immunotherapies. The authors showed the neoantigen-related mutations detected in nine of ten patients after ICI immunotherapies, but undetectable only in one patient, and suggested that activation of tumor-specific T cells might contribute to the response to ICI immunotherapies (80). However, it needs to validate the feasibility of personalized ctDNA testing with a large cohort in future studies to maximize the clinical outcomes of NSCLC patients in ICI immunotherapies.

## Conclusions

In conclusion, ctDNA testing is going to become a powerful approach applied in the clinical management of NSCLC patients at diagnosis, dynamic monitoring drug treatment or disease progression. However, it is still a great challenge because of the very low level of ctDNA released in the blood. In this regard, several ultra-sensitive and specific approaches were developed to detect somatic alterations by NGS-based ctDNA testing (36, 47, 81). In addition, several studies have shown tumor-derived DNA detected in other body fluids (82–84). Indeed, tumor-derived DNA from pleural effusion in patients with lung cancer was detected with high levels, suggesting that pleural effusion testing is an alternative and feasible method for mutations identification (82). However, more clinical trials are required for verifying these findings and for providing the standard operation procedure. Moreover, ctDNA testing has attracted great attention in early tumor detection of several common cancer types (57, 85–87), although it is still a long way to apply ctDNA testing in the clinical routine.

## Author Contributions

JY, BG, TM, and LL conceived the concept of the work. JY, YH, MZ, BJ, GT, and YG performed the experiments. YZ, JY, YH, and MZ wrote the paper. All authors contributed to the article and approved the submitted version.

## Funding

This research was funded by the Scientific Research Projects of the Inner Mongolian (no. 201702135), the Natural Science Foundation of Hunan province (no. 2018JJ3570), and the Project to Introduce Intelligence from Oversea Experts to Changsha City (no. 2089901).

## Conflict of Interest

JY, BJ, and GT were employed by Geneis Beijing Co., Ltd. BG and TM were employed by Genetron Health (Beijing) Co. Ltd.

The remaining authors declare that the research was conducted in the absence of any commercial or financial relationships that could be construed as a potential conflict of interest.

## Publisher’s Note

All claims expressed in this article are solely those of the authors and do not necessarily represent those of their affiliated organizations, or those of the publisher, the editors and the reviewers. Any product that may be evaluated in this article, or claim that may be made by its manufacturer, is not guaranteed or endorsed by the publisher.
